# Sporadic and genetic forms of paediatric somatotropinoma: a retrospective analysis of seven cases and a review of the literature

**DOI:** 10.1186/1750-1172-6-67

**Published:** 2011-10-24

**Authors:** Cécile Nozières, Pascale Berlier, Clémentine Dupuis, Catherine Raynaud-Ravni, Yves Morel, Françoise Borson Chazot, Marc Nicolino

**Affiliations:** 1 Fédération d'Endocrinologie du Pôle Est, Groupement Hospitalier Lyon Est, 69677, Bron, Cedex, France; 2 Service d'Endocrinologie Pédiatrique; Groupement Hospitalier Lyon Est, 69677, Bron, Cedex, France; 3 Service de Pédiatrie, CHU La Tronche, BP2178, 38243, Grenoble, Cedex 9, France; 4 Service de Pédiatrie, Hôpital Nord, 42055, Saint-Etienne, Cedex 2, France; 5 Service d'Endocrinologie Moléculaire et Maladies Rares, Centre de Biologie Est, Hospices Civils de Lyon, Bron, Cedex, France

## Abstract

**Background:**

Somatotropinoma, a pituitary adenoma characterised by excessive production of growth hormone (GH), is extremely rare in childhood. A genetic defect is evident in some cases; known genetic changes include: multiple endocrine neoplasia type 1 (MEN1*)*; Carney complex; McCune-Albright syndrome; and, more recently identified, aryl hydrocarbon receptor-interacting protein (AIP). We describe seven children with somatotropinoma with a special focus on the differences between genetic and sporadic forms.

**Methods:**

Seven children who presented in our regional network between 1992 and 2008 were included in this retrospective analysis. First-type therapy was somatostatin (SMS) analogues or transsphenoidal surgery. Control was defined as when insulin-like growth factor-1 (IGF-1) levels were within the normal range for the patient's age at 6 months after therapy, associated with decreasing tumour volume.

**Results:**

Patients were aged 5-17 years and the majority (n = 6) were male. Four patients had an identified genetic mutation (McCune-Albright syndrome: n = 1; MEN1: n = 1; AIP: n = 2); the remaining three cases were sporadic. Accelerated growth rate was reported as the first clinical sign in four patients. Five patients presented with macroadenoma; invasion was noted in four of them (sporadic: n = 1; genetic: n = 3). Six patients were treated with SMS analogues; normalisation of IGF-1 occurred in one patient who had a sporadic intrasellar macroadenoma. Multiple types of therapy were necessary in all patients with an identified genetic mutation (4 types: n = 1; 3 types: n = 2; 2 types: n = 1), whereas two of the three patients with sporadic somatotropinoma required only one type of therapy.

**Conclusions:**

This is the first series that analyzes the therapeutic response of somatotropinoma in paediatric patients with identified genetic defects. We found that, in children, genetic somatotropinomas are more invasive than sporadic somatotropinomas. Furthermore, SMS analogues appear to be less effective for treating genetic somatotropinoma than sporadic somatotropinoma.

## Background

Somatotropinoma is a pituitary adenoma characterised by excessive production of growth hormone (GH) from the pituitary gland [1]. In adulthood, somatotropinoma is associated with severe symptoms, including organomegaly and dysmorphia [2], and diagnosis is usually delayed by six years after the initial presentation of symptoms [3]. Somatotropinoma is rare in childhood and adolescence, and there is little literature on this topic. As in adults, diagnosis in children is delayed, but clinical presentation differs with accelerated growth being the first symptom observed after dysmorphic features [1].

Genetic defects have been recently identified in some somatotropinoma cases. These defects include multiple neoplasia syndromes, including the multiple endocrine neoplasia type 1 (MEN1) [4,5], the Carney complex [6] and McCune-Albright syndrome [7]. Recently, mutations in the aryl hydrocarbon receptor interacting protein gene (AIP) were identified in some patients with familial isolated pituitary adenoma (FIPA) [8,9]. Such mutations account for 15% of FIPA kindreds and are associated with somatotropinomas, prolactinomas, non-secreting adenomas and rare cases of Cushing disease [10]. We present data on all children developing a somatotropinoma before 18 years of age between 1992 and 2008 in our regional network for the management of paediatric patients with endocrine disorders. Four of the children presented with a known genetic defect. The aim of this work was to analyse the clinical, biological, radiological and therapeutic aspects of these adenomas.

## Methods

All children (n = 7) with a GH-secreting pituitary adenoma that was diagnosed before 18 years of age in three hospitals in France [Lyon (n = 5), Saint-Etienne (n = 1) and Grenoble (n = 1)] were included in this analysis. One case was diagnosed in 1992 (when the patient was six years of age), and the other six cases were diagnosed between 2005 and 2008. We excluded patients with GH-independent gigantism, such as Beckwith-Wiedemann syndrome or Sotos syndrome, and patients with GH-releasing hormone secreting tumours.

A diagnosis of excessive production of GH was established by a high level of serum GH over 24 hours, not suppressible to <1 μg/L, 180 minutes after a 75 g oral glucose tolerance test, or a high insulin-like growth factor-1 (IGF-1) level for the patient's age.

Magnetic resonance imaging (MRI) was performed in six cases; images were analysed by the same radiologists. Invasiveness on MRI was assessed using the Hardy-Vezina classification [11]. Tumours were considered as invasive when presenting with extrasellar expansion to the cavernous or sphenoidal sinus. For the oldest case, computed-tomography scanning was performed in 1992, and monitoring was done by MRI from 2000 onwards.

### Immunometric assay

Serum GH levels were measured by radioimmunometric assay calibrated to the WHO 98/574 standard. Before 1995, the GH assay (for one patient) was performed using a polyclonal antibody kit.

Serum IGF-1 levels were measured using the IGF-1 RIACT kit (CIS Bio International). Results were presented as standard deviation (SD), adjusted for sex, age and pubertal stage. Prolactin was measured by radioimmunometric assay calibrated on 84/500 standard. Other hormones of the pituitary axis were also studied.

### Treatment protocol

Somatostatin (SMS) analogues or transsphenoidal surgery were the first-type treatment, depending on each patient's clinical evolution and criteria. Patients received long-acting octreotide (10-30 mg) or lanreotide (30-120 mg), managed by the patient's clinician, according to IGF-1 level and growth curve. Patients also received cabergoline at a dose of 0.5 mg once to three times weekly.

Control was defined as when IGF-1 was within the normal range for the patient's age at six months after therapy, associated with a decreasing tumour volume of more than 30%. SMS analogue resistance was defined in the present study as an IGF-1 level of more than +2 SD at six months when receiving the maximum dose of octreotide or lanreotide.

## Results

Baseline clinical characteristics for the seven patients are presented in Table 1.

**Table 1:** Baseline clinical characteristics of patients with somatotropinoma

Patient	Sex	Date of diagnosis	Age at diagnosis (years)	Symptoms/reason for consultation	Size at diagnosis (SD)
1	Male	10/1992	6.5	Monitoring of McCune-Albright syndrome	Bone deformity, non-measurable size

2	Female	01/06/2007	6.5	Accelerated growth	+4

3	Male	29/06/2005	13	Accelerated growth	+2

4	Male	18/01/2006	16.5	Headaches, gynaecomastia	+3

5	Male	16/03/2006	5.5	Accelerated growth	+4

6	Male	2008	17	Acquired resistance to cabergoline	+0,5

7	Male	07/2006	9	Accelerated growth	+4

**Figure 1:**
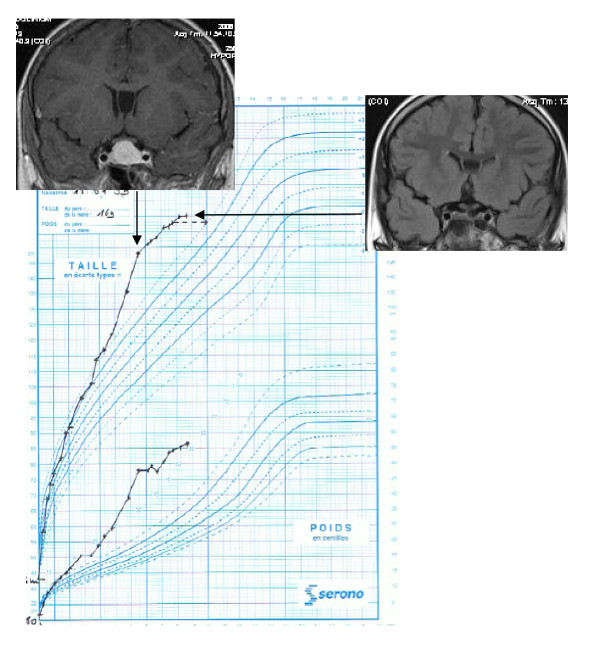
**Growth curve of patient 5**. At the time of diagnosis, magnetic resonance imaging (MRI) showed a non-invasive macroadenoma. Control was obtained with first-line somatostatin analogues. There was a correlation between decelerated growth, insulin-like growth factor-1 levels and disappearance of the macroadenoma on MRI.

**Figure 2:**
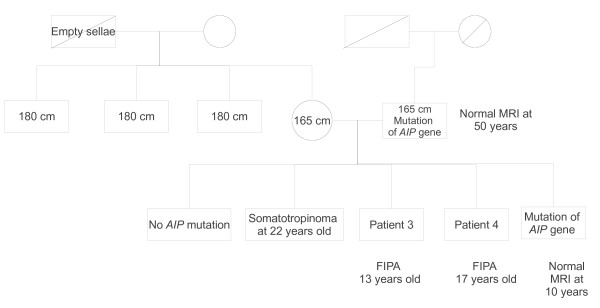
**Family history of patients 3 and 4**.

Four patients (patients 2, 3, 5 and 7) presented for a clinical consultation because of accelerated growth. (the growth curve for patient 5 is shown in Figure 1). The other patients presented for monitoring of McCune-Albright syndrome (patient 1), gynaecomastia (patient 4) or acquired resistance to cabergoline (patient 6). Acromegaly was diagnosed in patient 1, three months after the onset of McCune-Albright syndrome, with macrocrania, a cardiac anomaly, enlarged hands and feet and nocturnal snoring. Patients 3 and 4 both presented with headaches and visual disturbances. Patient 6 had been treated with dopamine agonists for a macroprolactinoma since ten years of age and GH excess was suspected because of an acquired resistance to cabergoline, headaches and enlarged hands and feet. Only patients 2, 3 and 4 presented with a family history of pituitary adenomas (the family history of patients 3 and 4 is shown in Figure 2). Genetic analysis was positive for MEN1 in patient 2 (intron 3, c.765-6C-> T-splicing site) and AIP in patients 3 and 4 (exon 5, c.718T-> C- missense mutation), diagnosed at age 6, 13 and 16 years of age, respectively. Extensive genetic analysis (MEN1, AIP, Carney) was negative in patients 5 and 7, who were diagnosed at five and nine years of age, respectively. MEN1 and AIP analyses were negative in patient 6. MRI revealed an invasive macroadenoma with suprasellar and lateral extension to the cavernous sinus in patients 2, 3, 4 and 6, a non-invasive intrasellar macroadenoma in patient 5 and a microadenoma in patient 7.

Therapeutic data for the seven patients are presented in Table 2 (FU: Follow-Up; C: Controlled; NC: Not Controlled).

**Table 2:** Therapeutic intervention and follow-up in patients with somatotropinoma

Patient	Genetic mutation	IGF1 (μg/L) and MRI at diagnosis	First-type therapy	Second-type therapy	Third-type therapy	Fourth-type therapy	Control of disease	Last IGF1 (μg/L) and MRI	F-U (Years)
1	MAS	406 (+3SD) Hyperplasia 565 (+4SD)	Octreotide LAR 30 mg (monthly- 18 months)	+ Cabergoline 1 mg/week	Pegvisomant 20 mg/day		C	333 (-1SD) NA	7
2	MENI	Invasive Macroadenoma (36 × 27 × 28 mm) 1259 (+4SD)	Trans-sphenoidal surgery	Octreotide LAR 30 mg monthly (6 months - growth of adenoma with visual disturbance; 3 surgeries)	+ Cabergoline with visual (1 mg/week)	Proton therapy	NC	689 (+2.5SD) 14 × 10 mm (-68%)	3
3	AIP	Invasive Macroadenoma (21 × 26 mm) 997 (+4SD)	Lanreotide LAR 90 mg monthly (6 months)	Octreotide LAR 30 mg monthly + cabergoline (1 mg/week)	Transphenoidal surgery		NC	1184 (+4SD) 10 × 12 × 9 mm (-50%)	4
4	AIP	Invasive Macroadenoma (16 × 21 × 11 mm) 934 (+4SD)	Lanreotide LAR 60 mg monthly (6 months)	Transphenoidal surgery			C	451 (0SD) No visible adenoma	6
5	Negative genetic analysis (Carney, MEN1, AIP)	Non invasive macroadenoma (19 × 14 × 20 mm)	Lanreotide LAR 60 mg monthly				C	282 (+1SD) No visible adenoma	4
6	Negative genetic analysis (MEN1, AIP)	777 (+3SD) Invasive macroadenoma (26 × 26 × 32 mm)	Octreotide LAR 30 mg monthly (6 months) added to the previous Dopamine agonist treatment (Cabergoline 1,5 mg/week)				NC	717(+3SD) 34 × 21 × 23 mm (+6%)	2
7	Negative genetic analysis (Carney, MEN1, AIP)	574 (+4SD) Microadenoma (7,6 × 9 × 7,5 mm)	Trans-sphenoidal surgery				C	504 (+1,5 SD) No visible adenoma	4

**Figure 3:**
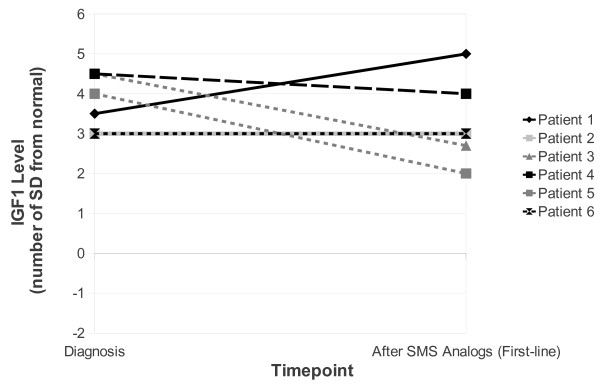
**Change in insulin-like growth factor-1 levels during somatostatin (SMS) analogue therapy**. Only patient 5, with intrasellar macroadenoma and a sporadic form, was controlled after first-line SMS analogues.

Transsphenoidal surgery was the first-type therapy in patient 2 (presenting with visual defects due to chiasmatic compression) and patient 7 (presenting with a sporadic microadenoma). Patient 7 was cured but patient 2, with MEN1 and an invasive macroadenoma, remained uncontrolled despite multiple types of treatment (Table 2). Overall, six patients were treated with SMS analogues; in 5 patients (1, 3, 4, 5 and 6), this comprised first-type therapy. As shown in Figure 3, normalisation of IGF-1 occurred in only one case of sporadic intrasellar macroadenoma (patient 5). In this patient, an impressive tumoural response was observed with normalization of MRI after 33 months. In the other five cases, four of them being of genetic form, IGF-1 was not normalized, requiring additional types of therapy (3 types: n = 1; 2 types: n = 2; 1 type: n = 1).

At the end of treatment, four patients had normal IGF-1 levels for their age, sex and pubertal stage (Figure 3, Table 2). Normalization of IGF-1 levels occurred in two out of the three patients with a sporadic form (patients 5 and 7) who received only one type of therapy and two out of the four patients with a genetic form who received 2-3 types of therapy. Disease control was not achieved in patients 2 and 3 and 6. A decrease in growth was observed when disease control was achieved (Figure 1).

## Discussion

This series of paediatric patients is one of the largest series of somatotropinomas in children and gives new information on the therapeutic management of this disease. Recent epidemiological data have shown that the overall prevalence of pituitary adenomas was greater than previously estimated. It was measured at 94.4 and 77.6/100,000 inhabitants in the and Belgium [12] and United Kingdom [13] respectively, with somatotropinomas representing 13% and 11% of pituitary adenomas [12,13]. However, epidemiological data remain scarce in children. In accordance with our results, the literature reports a higher prevalence of GH-secreting pituitary adenomas in boys than in girls [14-19]. This may be particularly true for genetic forms, and particularly in patients with AIP-related pituitary adenomas. Daly et al. showed recently in a series of 96 AIP patients that 63.5% of them were male [10]. In contrast, in a series of 83 *a priori *sporadic somatotropinomas in adults and young patients, Tichomirowa et al. [20] observed that only 44.5% were male. However, an AIP mutation was identified in eleven cases (13.3%), and eight of them were men.

The presentation of somatotropinoma in children differs according to whether the epiphyseal plate has fused or not. In the former situation, symptoms are similar to those seen in acromegalic adults [1]. In contrast, if somatotropinoma occurs prior to fusion of the epiphyseal plate, accelerated growth ensues with large size and extreme "gigantism" [1]. In our series, four patients had accelerated growth, two of whom had genetic forms of somatotropinoma. Daly et al. showed that in adult patients with AIP-related pituitary adenoma, gigantism was significantly more frequent than in controls (32% vs. 6.5%; p < 0.000001) [10]. In our series, whatever the age at diagnosis, the majority of patients presented with enlarged hands and feet.

Most pituitary adenomas in childhood are macroadenomas, and locally invasive tumours are frequently observed [15,21]; headaches and visual disturbances are, therefore, common [15,21,22]. In our series, five of seven patients presented with macroadenoma; of these patients, four had invasive macroadenomas, and three of these four had a genetic mutation (one MEN1, two AIP). This is consistent with the literature in adult patients, which reports larger tumours and a more aggressive presentation in patients with MEN1 versus without MEN1 (85% vs. 42%; p < 0.001) [4]. Recently, Daly et al. showed that the maximum tumour diameter was higher in patients with AIP-related somatotropinoma adenoma (n = 75) than in age-matched AIP-negative patients (n = 232) (22.5 [7.0-60] vs. 16.0 [3.0-48] mm; p < 0.00026) [10].

Moreover, four studies reported a high proportion of genetic forms in children and young subjects [20,23-25]. Three of them [23-25] included, as did our study, seven cases of acromegaly in young subjects, with identification of a genetic form in 2-4 cases. In the fourth study, conducted by Tichomirowa et al., which included 39 patients with a pituitary macroadenoma diagnosed before 18 years, an AIP mutation was identified in 20.5% of cases [20].

In both adults and children, conventional treatment of somatotropinoma is transsphenoidal surgery [15,18]. In cases of intracavernous extension, or when surgery has been incomplete, pre- and/or post-operative SMS analogue treatment may be administered [16,26] and has been shown to be safe in paediatric patients [14]. The GH receptor antagonist pegvisomant, or cabergoline in cases of somato-prolactinomas, can be used in acromegalic patients who have failed to respond to conventional treatment [14,27]. In our region, SMS analogues were effective in one of the six cases in whom they were used.

Cabergoline was administered to two patients in our series (both with genetic somatotropinoma) as second-type therapy in combination with SMS analogues. One of these patients required no further therapy, while the other (who had McCune-Albright syndrome) was switched to pegvisomant, which successfully reduced IGF-1 levels to -1 SD, consistent with the literature [28,29].

In adults, genetic forms of somatotropinoma are more difficult to control than sporadic forms. In a study of adult patients with pituitary adenomas, Verges et al. observed a normalization of pituitary hypersecretion in 90% of patients with sporadic adenomas and 42% of patients with MEN1 genetic alterations [4]. To date, little is known on the therapeutic response in children. Personnier et al., in a recent review, observed that GH control was obtained in 69% of cases in children [30]; in that analysis, genetic and sporadic forms were analyzed together. Accordingly, in another series of eleven sporadic paediatric pituitary adenomas, six were considered to be controlled by SMS analogues before and after surgery. Tumour size was reduced by 51.0% overall [14]. In contrast, Tichomirowa et al. [20] observed that, in a series of eleven paediatric and young patients with an AIP-related somatotropinoma, at least two surgical interventions were necessary in 36.4% of cases, and that post-operative SMS analogue therapy was only effective in 11% of patients [20]. In our series, control was achieved in two out of the three patients with a sporadic somatotropinoma after one type of therapy. In contrast, all patients with genetic somatotropinoma required two (n = 2) or three (n = 2) types of therapy, and only two out of four such patients were cured. Three patients received SMS analogues as first-type therapy; these were followed by cabergoline, pegvisomant or transsphenoidal surgery. In the fourth patient, transsphenoidal surgery was ineffective as first-type therapy, and IGF-1 levels remained +2 SD even after subsequent SMS analogues and radiotherapy. These findings suggest that SMS analogues have little efficacy in patients with genetic somatotropinoma and that, as reported in adults [4], hormonal control is more difficult to achieve in patients with genetic somatotropinomas than in patients with sporadic forms.

## Conclusions

Somatotropinomas in children and adolescents are rare and more aggressive than those seen in adults. In this small series of patients, genetic somatotropinomas were more invasive than sporadic somatotropinomas. Our data suggest that SMS analogues, while effective in sporadic somatotropinoma, are less effective in genetic somatotropinoma. Further research on the incidence and types of genetic mutations in children and adolescents with somatotropinoma is necessary to increase our understanding of the disease. In addition, studies of different therapeutic options in genetic somatotropinoma would provide more information about why SMS analogues are less effective in these patients, and allow for the development of new treatment strategies in this setting.

## Competing interests

The authors declare that they have no competing interests.

## Authors' contributions

CR-R, CD, PB: each of them provided data from a patient. YM measured and analyzed IGF-1 and prolactin levels. FB-C provided data from three patients and managed this work. MN provided data from a patient and managed this work. CN collected and analyzed all data and wrote this paper with the assistance of Claire Byrne of *in*Science Communications (a Wolters Kluwer business). All authors read and approved the final manuscript.
